# Supplementary material for: All-at-once RNA folding with 3D motif prediction framed by evolutionary information
Source: Nat Methods. 2025 Oct 3;22(10):2094–106. doi: 10.1038/s41592-025-02833-w (PMC12510878; doi:10.1038/s41592-025-02833-w)
Supplement: Supplementary file 3 — Source code to run CaCoFold-R3D. Data and results. [file 41592_2025_2833_MOESM3_ESM.gz › supplemental_material/results/CaCoFold-R3D/CaCoFold-R3D_Rfam_vshuffle/RF01515_Afu_514.R2R.sto.pdf]

RF01515_Afu_514

5′-U ●●● A U G A U G C G G U A A U U U G C G R U U G A Y ● C R ● Y ●● C U G U G A A ○ C Y C Y C G C C R G R G U U Y Y Y Y ●● A G C A R Y ● Y U ●●●●● R U G A U A U U C A U U U C U U A C A U A U U C U G A ●●
